# Amyotrophic lateral sclerosis in a tricenarian female

**DOI:** 10.1016/j.radcr.2024.10.153

**Published:** 2024-11-30

**Authors:** Anshul Sood, Gaurav Vedprakash Mishra, Pallavi Kar, Shreya Khandelwal, Shubhi Gaur, Nishtha Manuja

**Affiliations:** a Deptartment of Radiodiagnosis, Jawaharlal Nehru Medical College, Datta Meghe Institute of Higher Education and Research, Sawangi (Meghe), Wardha, Maharashtra, India 442001; b Deptartment of Medicine, Jawaharlal Nehru Medical College, Datta Meghe Institute of Higher Education and Research, Sawangi (Meghe), Wardha, Maharashtra, India 442001

**Keywords:** MRI, Radiology, Wine glass sign, ALS, Female

## Abstract

Amyotrophic lateral sclerosis (ALS) is a motor neuron disease characterized by the progressive degeneration of the upper and lower motor neurons. This disease is mostly observed in patients of the 6th decade or above, and it is extremely rare to observe this pathology in patients less than 50 years of age. This manuscript depicts the magnetic resonance imaging findings of ALS showing a wine glass sign in a 31-year-old female from a rural area with complaints of progressive limb weakness and muscle wasting.

## Introduction

Amyotrophic lateral sclerosis (ALS), Lou Gehrig's, or Charcot disease, is a rare central nervous system pathology affecting both the upper and lower motor neurons. Pathologically, it causes the degeneration of the anterior horn cells and upper motor neurons by affecting the Betz cells in the primary cortex with secondary Wallerian degeneration [1,2]. The affected individual presents with complaints related to both upper and lower motor neurons, which, along with history and clinical examination, helps to make the diagnosis. Magnetic Resonance Imaging (MRI) of the brain is the primary imaging modality of choice, which shows a T2-hyperintense signal in the affected corticospinal tracts and aids in confirming the diagnosis and ruling out another differential diagnosis. The prognosis of the patient is bad, with demise within 2-6 years [3,4].

## Case report

**Fig. 1:**
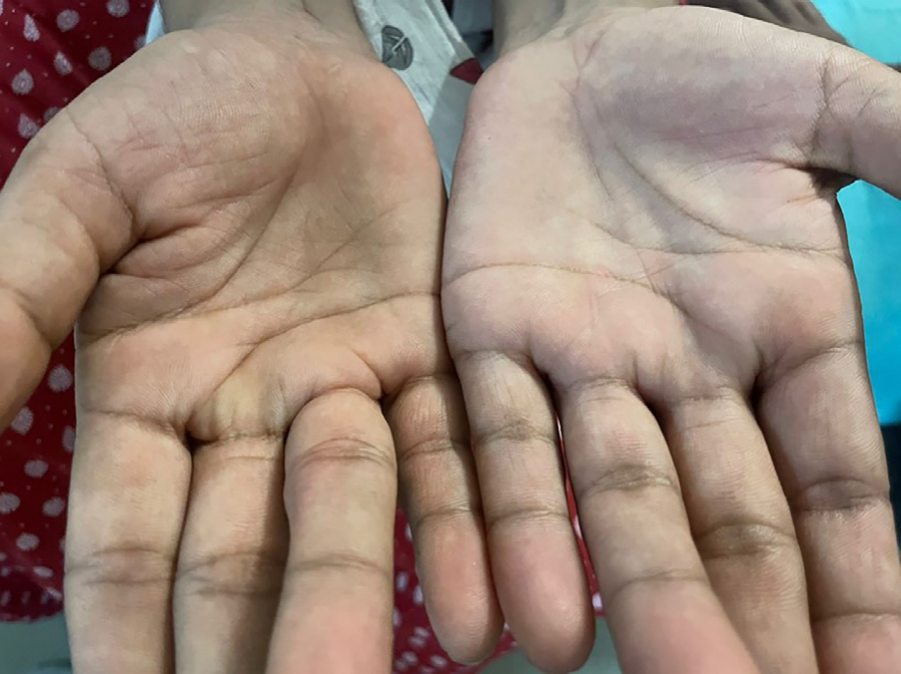
Clicked picture of the hands showing muscle wasting.

A 31-year-old female patient from a rural area in India was brought to the neurology outpatient department (OPD) with her husband for complaints of slurring of speech for the past 2 years, progressive difficulty in walking, and weakness in both upper and lower limbs for the past year. The limb weakness had progressed to the stage where she was unable to perform daily activities. The patient does not have any known systemic illness. She has not undergone any surgical procedures to date. The family history is insignificant, with no known similar cases in the first and second-degree relatives. The examination revealed a weak hand grip for both hands with an increased muscle tone and muscle wasting, as shown in Fig. 1. Routine blood investigations did not reveal anything significant.

**Fig. 2:**
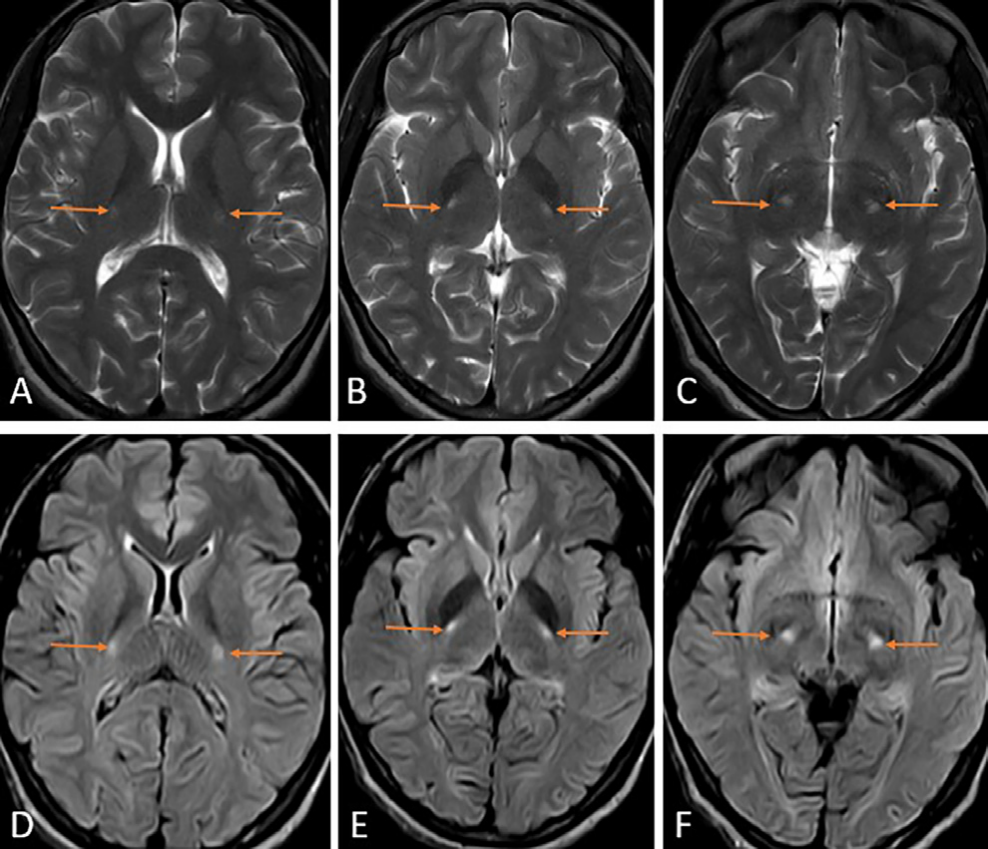
Magnetic Resonance Imaging of the brain axial sections T2 weighted imaging (A, B, C), fluid attenuation recovery sequence (D), (E), and (F) showing hyperintense signal involving bilateral corticospinal tracts, bilateral internal capsule, extending superiorly up to the motor cortex and inferiorly up to the cerebral peduncles of the midbrain (orange arrows) suggesting amyotrophic lateral sclerosis.

**Fig. 3:**
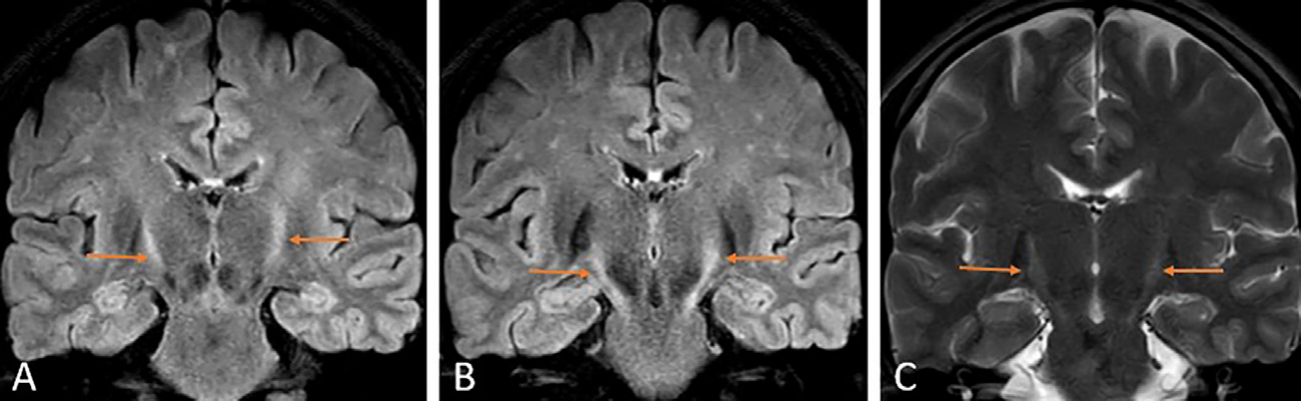
Magnetic Resonance Imaging of the brain coronal sections fluid attenuation recovery sequence (A, B), and T2 weighted imaging (C) showing hyperintense signal involving bilateral corticospinal tracts, bilateral internal capsule, extending superiorly up to the motor cortex and inferiorly up to the cerebral peduncles of the midbrain showing wine glass sign (orange arrows) suggesting amyotrophic lateral sclerosis.

Imaging of the brain was conducted using magnetic resonance imaging (MRI), which revealed T2 weighted imaging and fluid attenuation recovery (FLAIR) sequence hyperintensity in the corticospinal tracts, posterior limb of internal capsule extending to involve the subcortical white matter superiorly, and cerebral peduncles of the midbrain inferiorly suggesting a diagnosis of amyotrophic lateral sclerosis as shown in Fig. 2, Fig. 3.

The patient was admitted and was managed symptomatically. The patient was started with physiotherapy exercises of both upper and lower limbs twice daily. Also, speech therapy was done daily. The patient was advised for the treatment with intravenous Riluzole, but it was refused due to financial constraints. After 10 days, the patient was discharged with advice to follow up after 15 days or if any complaints develop.

## Discussion

Amyotrophic lateral sclerosis (ALS), also known as Charcot disease and Lou Gehrig disease, is a rare disease with an incidence of 0.78 per 1,00,000 population in a year. It is most commonly seen in males in the 6th decade, with an average age of onset being 58-60 years. It is extremely rare to observe ALS before 50 years. The time from the onset of symptoms to death is almost 3-6 years [1,2,3]. The disease involves damage to both upper and lower motor neurons in the brainstem and primary motor cortex, which causes progressive muscle weakness, leading to death due to respiratory insufficiency. The term lateral sclerosis signifies the degeneration of the pyramidal tracts [4]. Pathologically, the disease is manifested by the death of cortical Betz cells (upper motor neurons) [5].

The symptoms with which the patient presents include the signs of both upper and lower motor neuron degeneration marked by weakness of the forearm and hands, muscle wasting of limbs and diaphragm, leg spasticity, and generalized hyperreflexia. However, the sensory and intellectual functions are preserved. The differential diagnosis includes other degenerative motor neuron diseases, including multiple sclerosis, conus lesions, compressive cervical myelopathy, and cerebrovascular disease, which are ruled out using lab tests and imaging [6,7].

The imaging modality of choice is MRI, which reveals T2WI/FLAIR hyperintensities in the bilateral corticospinal tracts extending from the brainstem to the centrum semiovale, giving a wine glass sign in the coronal sections [8]. Krabbe disease, X-linked Charcot-Marie tooth disease, and adrenomyeloneuropathy also have similar imaging findings. However, the clinical complaints differ from those of ALS. Some cases have reported T2WI hypointensity in the precentral cortex, suggesting changes in the anterior subcortical white matter [9]. Association of mild cerebral atrophy [10], hyperintensities in anterolateral columns of the cervical cord [11] with ALS have been previously reported in the literature. Diffusion tensor imaging shows an increase in the mean diffusivity values and a decrease in fractional anisotropy values along the corticospinal tracts [12]. MR spectroscopy reveals a decrease in N-acetyl aspartate and glutamate with an increase in the choline and myoinositol [5].

The treatment is symptomatic, with a survival time of 3-6 years from the onset of the disease. The death is mostly due to respiratory complications. The patient survival time has been observed to be increased by the use of a few therapies, including riluzole, which is a glutamate antagonist [13,14].

## Patient consent

An informed verbal and written consent was obtained from the patient.
